# Bacteria from gut microbiota associated with diarrheal infections in children promote virulence of Shiga toxin-producing and enteroaggregative *Escherichia coli* pathotypes

**DOI:** 10.3389/fcimb.2022.867205

**Published:** 2022-08-09

**Authors:** Mariana Izquierdo, Joaquín Lopez, Pablo Gallardo, Roberto M. Vidal, Juan C. Ossa, Mauricio J. Farfan

**Affiliations:** ^1^ Departamento de Pediatría y Cirugía Infantil, Facultad de Medicina, Universidad de Chile, Santiago, Chile; ^2^ Programa de Microbiología y Micología, Instituto de Ciencias Biomédicas, Facultad de Medicina, Universidad de Chile, Santiago, Chile

**Keywords:** Shiga toxin-producing *Escherichia coli*, enteroaggregative *Escherichia coli*, gut microbiota, inflammation, diarrheagenic *E. coli*

## Abstract

**Background:**

Diarrheagenic *E. coli* (DEC) pathogenicity relies on the interaction of bacteria with the host’s gut environment, which is regulated by the resident microbiota. Previously, we identified indicative bacterial species of gut microbiota in DEC-positive stool samples from children. Here, we evaluated the role of two indicative species, *Citrobacter werkmanii* (CW) and *Escherichia albertii* (EA), in the virulence of two DEC pathotypes, Shiga toxin-producing (STEC) and enteroaggregative (EAEC) *Escherichia coli*.

**Methods:**

We determined the effect of supernatants obtained from CW and EA cultures on the gene expression of STEC strain 86-24 and EAEC strain 042 by RNA-seq analysis. We evaluated IL-8 secretion from T84 cells infected with these DEC strains in the presence or absence of the supernatant from EA. The effect of the supernatant from EA on the growth and adherence of STEC and EAEC to cells was also evaluated. Finally, we studied the effect of the EA supernatant on the STEC-induced inflammation mediated by the long polar fimbriae (Lpf) in T84 cells and the expression of plasmid-encoded toxin (Pet) in EAEC.

**Results:**

RNA-seq analysis revealed that several virulence factors in STEC and EAEC were upregulated in the presence of supernatants from CW and EA. Interestingly, an increase in the secretion of IL-8 was observed in cells infected with STEC or EAEC in the presence of a supernatant from EA. Similar results were observed with the supernatants obtained from clinical strains of *E. albertii*. The supernatant from EA had no effect on the growth of STEC and EAEC, or on the ability of these DEC strains to adhere to cells. We found that Pet toxin in EAEC was upregulated in the presence of a supernatant from EA. In STEC, using mutant strains for Lpf fimbriae, our data suggested that these fimbriae might be participating in the increase in IL-8 induced by STEC in cells in the presence of a supernatant from EA.

**Conclusion:**

Supernatant obtained from an indicative species of DEC-positive diarrhea could modulate gene expression in STEC and EAEC, and IL-8 secretion induced by these bacteria. These data provide new insights into the effect of gut microbiota species in the pathogenicity of STEC and EAEC.

## Introduction

Diarrheagenic *Escherichia coli* (DEC) are the most common bacterial cause of diarrhea in both developing and industrialized regions, primarily affecting children under 5 years of age (Gomes et al., 2016). According to the detection of the virulence factors, six classical categories or pathotypes have been described (Kaper et al., 2004). DEC pathogenesis comprises three stages: adherence and colonization, production of toxins, and diarrhea followed by inflammation (Croxen et al., 2013).

Shiga toxin-producing *Escherichia coli* (STEC) initial attachment to intestinal cells is mediated by adhesins, such as the long polar fimbriae (Lpf) (Torres et al., 2002). Intimate attachment is defined by the ability of STEC to induce characteristic attaching and effacing lesions on intestinal epithelia, which is mediated by proteins encoded by genes on the pathogenicity island called locus of enterocyte effacement (LEE) (Stevens and Frankel, 2014). Enteroaggregative *Escherichia coli* (EAEC) pathogenesis is regulated by a plasmid-encoded virulence regulator called AggR. This master regulator controls the expression of several chromosome- and plasmid-encoded genes, including aggregative adherence fimbriae (AAFs), a key virulence factor in EAEC pathogenesis responsible for intestinal cell adherence, and dispersin, with its dedicated type I secretion system (Harrington et al., 2006; Morin et al., 2013). Another important virulence factor in EAEC is plasmid-encoded toxin (Pet), a protease that induces cytotoxic and cytopathic effects on different cultured cell lines by spectrin and fodrin degradation (Villaseca et al., 2000).

STEC and EAEC infections are characterized by acute inflammation of the colonic mucosa, where these DEC pathotypes induce the secretion of several pro-inflammatory markers, such as interleukin-8 (IL-8), as a response to the activation of the MAPK, AP-1, and NF-kB signaling pathways (Steiner et al., 1998; Dahan et al., 2002; Miyamoto et al., 2006). The mechanism of pathogenesis for DEC pathotypes and other enteropathogens involves a variety of interactions between bacterial and host factors (Kitamoto et al., 2016). Under well-defined environmental conditions, expression of virulence genes at the infection site occurs, allowing the bacteria to adhere and colonize the target cells (Barnett Foster, 2013). For STEC and EAEC, virulence gene expression is not completely understood, and most studies have focused on deciphering the molecular mechanism occurring inside the bacteria, while very little is known about the environmental factors that regulate pathogenesis at a specific time or place (Jubelin et al., 2018). Considering that the expression of virulence factors in bacterial pathogens is strongly regulated by environmental conditions at the infection site, it is not surprising that gut microbiota might play a role in the regulation of pathogenic mechanisms (Baumler and Sperandio, 2016; Jubelin et al., 2018). Evidence supporting the role of specific strains from the normal gut microbiota in STEC virulence regulation has been published (Kendall et al., 2012; Pacheco and Sperandio, 2012; Curtis et al., 2014; Pacheco and Sperandio, 2015). In a previous publication, we identified two indicative bacterial species of gut microbiota significantly associated with DEC-positive diarrhea stool samples from children under 5 years of age, *Citrobacter werkmanii* (*C. werkmanii*) and *Escherichia albertii* (*E. albertii*) (Gallardo et al., 2017). In this work, we evaluated the role of these indicative species in the pathogenicity of STEC and EAEC.

## Materials and methods

### Bacterial strains

STEC O157:H7 strain 86-24, EAEC O44:H18 strain 042, *C. werkmanii* (DSM-17579), and *E. albertii* (DSM-17582) were used in this study. The STEC mutant strain in Lpf fimbriae (STEC AGT210; Torres et al., 2004), the EAEC mutant strain in Pet toxin (EAEC 042*Δpet;* Betancourt-Sanchez and Navarro-Garcia, 2009) and *E. coli* HB101 were included in additional experiments. Five strains of *E. albertii* obtained from Brazilian patients, kindly provided by Tânia A. T. Gomes (Departamento de Microbiologia, Imunologia e Parasitologia, Escola Paulista de Medicina, Universidade Federal de São Paulo, São Paulo, Brazil), were also used. All strains were grown overnight in Luria-Bertani (LB) broth at 37°C and 180 rpm.

### CW and EA supernatant preparation

*C. werkmanii* and *E. albertii* strains were grown overnight at 37°C and 180 rpm in M9 minimal medium or LB broth. Bacteria were centrifuged at 5,000 rpm for 10 min and the supernatant was recovered and filtered with a 0.2 µm pore size filter. These filtered supernatants were considered 1× concentrations and were diluted 1:2 and 1:4 with fresh medium from the respective growth medium (M9 medium or LB broth) to obtain 0.5× and 0.25× dilutions, respectively.

### Cell lines

Human colonic T84 intestinal epithelial cells (CCL-248 ATCC) were routinely maintained in Dulbecco’s modified Eagle’s medium (DMEM)–F-12 medium, supplemented with 10% fetal bovine serum (FBS), penicillin (10 U/ml), and streptomycin (10 µg/ml), at 37°C in 5% CO_2_.

### RNA-seq and real-time PCR

After overnight growth, STEC and EAEC cultures were diluted 1:50 in DMEM High Glucose (DMEM HG) medium and left to grow for ~3 h at 37°C and 180 rpm, until the bacteria reached an optical density measured at 600 nm (OD_600nm_) equal to 0.3. Bacterial cultures were centrifuged at 5,000 rpm for 10 min and the supernatant was removed. The pellet was resuspended in the supernatant from *C. werkmanii* or supernatant from *E. albertii* prepared in M9 medium. As a control condition, STEC and EAEC were resuspended in M9 medium. These preparations were incubated for 3 h at 37°C and 150 rpm. The bacteria were centrifuged at 5,000 rpm for 10 min and the pellet was resuspended in TRIzol™ Reagent and stored at −80°C. RNA was extracted and incubated with DNase I to remove DNA contamination as previously described (Yanez et al., 2016). RNA integrity was evaluated using an agarose gel 2%, and then samples were sent to Macrogen in Korea for RNA sequencing (RNA-seq) and analysis. For the expression profiling analysis, the HTseq Python package was used. The expression profile was calculated for each sample, and the genes that satisfied |fc| ≥ 2 conditions compared to the control (DEC strains incubated with M9 medium) were considered differentially expressed genes.

To confirm the RNA-seq results, we evaluated the expression of the mainly virulence genes present in STEC (*ler*, *lpfA*, and *stx2*) and EAEC (*aggR*, *aafA*, and *pet*) by RT-qPCR for two replicated experiments. RNA was reverse-transcribed using the Transcriptor First Strand cDNA Synthesis Kit (Roche), and synthesized cDNA was used to quantify the expression of previously mentioned virulence factors using primers indicated in **Supplementary Table 1** (Brukner et al., 2015, Hinthong et al., 2015, Qu et al., 2014, Walters and Sperandio, 2006). All data were normalized using *16S* and *rpoA* genes as the endogenous control and analyzed using the comparative critical threshold (Ct) method (Yanez et al., 2016). Virulence gene expression was presented as fold changes over the expression level of DEC strains incubated with M9 medium.

### Growth curve

After overnight growth, STEC or EAEC cultures were diluted 1:50 in DMEM HG medium supplemented with 20% of *E. albertii* supernatant prepared in LB. Different concentrations of *E. albertii* supernatant (0.25×, 0.5×, and 1× dilutions in LB broth) were used. For the control condition, we used DMEM HG medium supplemented with 20% of LB broth. These bacteria were grown in agitation at 37°C for 6 h. OD_600nm_ was measured every 30 min using the Synergy HT reader (Biotek). These experiments were done in triplicate.

### T84 infection assay

Confluent T84 cells, grown in 24-well plates, were incubated for 30 min with 500 μl/well of DMEM F12 without antibiotics. Then, bacteria (EAEC 042, STEC 86-24, or STEC AGT210) were added to the monolayer with a multiplicity of infection (MOI) of 10. At the same time, 100 µl of different dilutions of *E. albertii* or *C. werkmanii* supernatant prepared in LB broth was added. As a control, 100 µl of LB broth was used. The plates were incubated at 37°C in 5% CO_2_ for 3 h and then washed three times with PBS. After we removed the PBS, cells were used to evaluate the inflammatory response to DEC infection or DEC adherence. Both experiments were done in triplicate

To evaluate the inflammatory response, 500 µl/well of DMEM-F12 with gentamicin 50 µg/ml were added to each well, and plates were incubated at 37°C in 5% CO_2_ for 3 h. After incubation, the supernatant was recovered and stored at −20°C. The level of IL-8 in the medium recovered from the infected cell was evaluated by ELISA, as previously described (Harrington et al., 2005). To evaluate DEC adherence, cells were lysed with a solution containing 0.5% (v/v) Triton X-100/PBS, and serial dilutions of the lysates were plated on LB agar. The number of adherent bacteria was determined by counting colony-forming units (CFU).

### Pet secretion assay

EAEC 042 and EAEC 042*Δpet* overnight culture were diluted 1:20 in LB broth and left to grow at 37°C and 160 rpm until OD_600nm_ equal to 0.9 was reached. Subsequently, the EAEC 042 culture was divided into five tubes, centrifuged at 4,500 rpm for 10 min and the supernatant was discarded. At the same time, *E coli* HB101, EAEC 042*Δpet, E. albertii* DSM-17582, and the clinical strain *E. albertii* 0621 overnight culture, prepared in a solution with LB : PBS in a proportion 1:2, were used to obtain the supernatant from these bacteria. Preliminary experiments showed that the incubation of EAEC 042 with LB : PBS in a proportion 1:2 had the best results to detect Pet toxin by Western blot (data not shown), selecting this condition to prepare the supernatants. The different supernatants were mixed with 1 ml of fresh LB broth and added to EAEC 042 bacteria. As a control, the supernatant from *E. albertii* DSM-17582 was also added to EAEC 042*Δpet* bacteria. The bacteria were incubated for 3 h at 160 rpm at 37°C, and OD_600nm_ was registered for further normalization. Bacterial cultures were centrifuged at 4,500 rpm for 15 min and the supernatant was recovered for Pet detection. Pet was precipitated with TCA and the pellet was resuspended with Tris-HCl/pH 8.8. Samples were analyzed on a 10% SDS-PAGE gel and dyed with Coomassie Blue. The amount of protein added on the gel was normalized by the previously registered OD_600nm_. For Western blotting, gels were transferred to a nitrocellulose membrane, and blotting membranes were incubated for 1 h in a blocking buffer with rabbit polyclonal anti-Pet antibodies (1:800 dilution). Following a washing step, membranes were incubated for 1 h in blocking buffer with an HRP-conjugated goat anti-rabbit IgG antibody (1:2,000 dilution). HRP was detected with the Western blotting reagent luminol (Santa Cruz Biotechnology). The digital images were analyzed using the IMAGE STUDIO LITE program.

### Statistical analysis

For T84 cell infection assays and growth curves, values are expressed as the means ± the standard errors for one of three experiments in triplicate. For these experiments, statistical significance between groups was analyzed using ANOVA test followed by Bonferroni’s multiple comparisons test using the Prism 6 software. A *p*-value <0.05 was considered statistically significant.

## Results

### STEC and EAEC virulence genes are modulated by supernatants obtained from *E. albertii* or *C. werkmanii* cultures

We evaluated the effect of the supernatant obtained from *E. albertii* (EA SP) and *C. werkmanii* (CW SP) cultures on the pathogenesis of STEC strain 86-24 and EAEC strain 042. To evaluate changes in gene expression, we incubated both STEC and EAEC individually, with EA or CW SP, and bacterial RNA was analyzed by RNA-seq.

**Figure 1:**
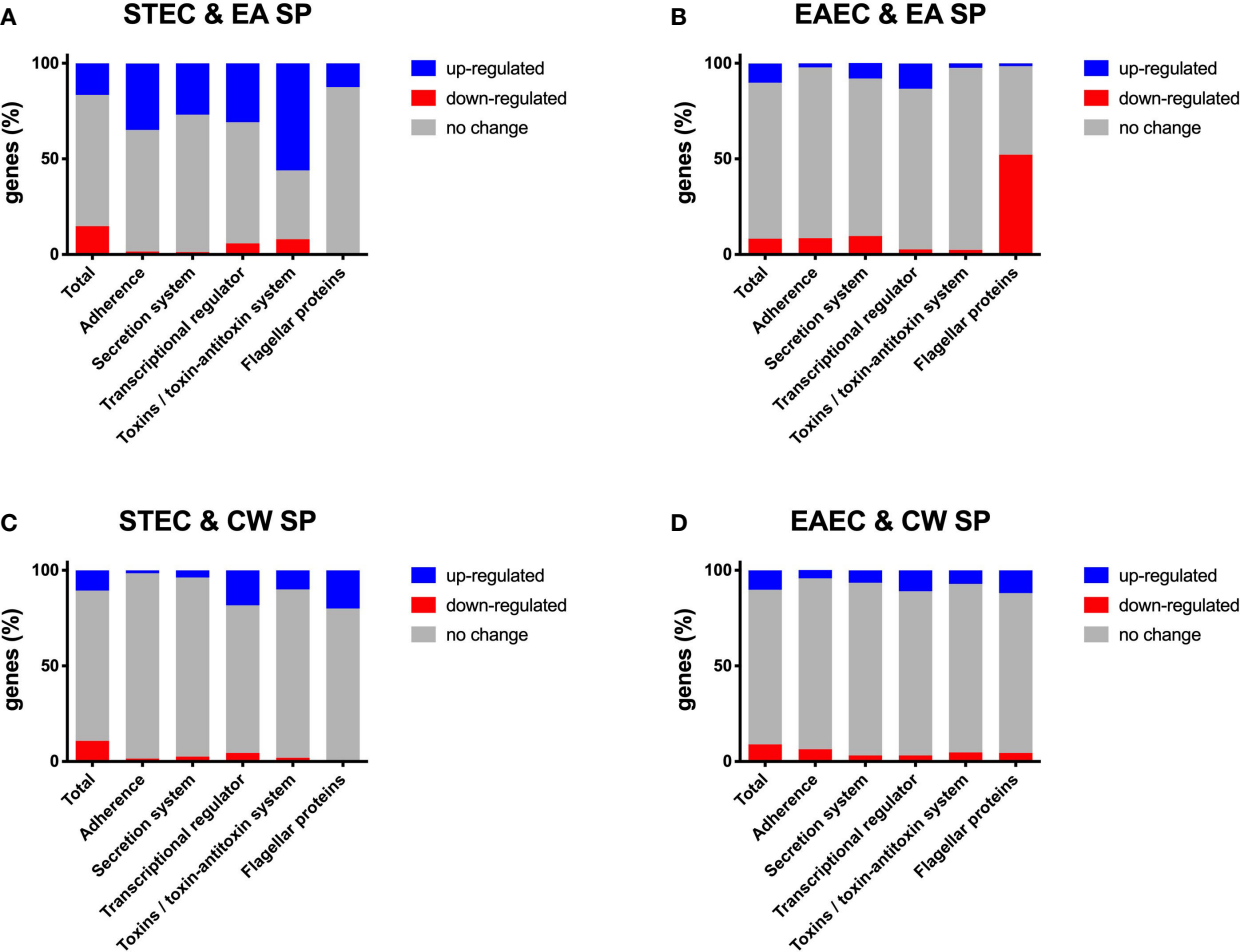
Changes in gene expression, grouped by category, for DEC pathotypes incubated with EA SP or CW SP. Percentage of genes in STEC **(A, C)** and EAEC **(B–D)** where a change was noted in gene expression after incubation with EA SP **(A, B)** or CW SP **(C, D)** compared to incubation with M9 medium. Changes observed in gene expression were organized according to their role during the pathogenic process.

After incubation with EA SP, we observed that 889 (16.7%) genes were upregulated and 780 (14.6%) genes were downregulated in STEC, whereas in EAEC, 525 (10.1%) genes were upregulated and 427 (8.3%) genes were downregulated (**Supplementary Table 2** and **Supplementary Figures 1A, B**). When we analyzed changes in gene expression for those involved in pathogenicity, for STEC, we found an upregulation of 35% of genes coding for proteins involved in adherence, 27% in secretion systems, 31% genes coding a transcriptional regulator, 56% of genes coding for toxins and toxin/antitoxin systems, and 12.5% of genes coding for protein responsible of STEC motility (**Figure 1A**). Changes for the main virulence factors in STEC are noted in **Table 1**. We observed that the gene coding for the master regulator Ler (*ler* gene) was upregulated ~4 times with EA supernatant compared to the control condition. Interestingly, we observed an upregulation in four genes of the operon coding for Lpf fimbriae, where gene expression of fimbrial subunit *lpfA*, *lpfD*, and *lpfE* increased 17.7, 2.7, and 2.9 times, respectively; and chaperone *lpfB* was upregulated 4.1 times. In addition, we observed an upregulation in genes coding for type I fimbriae (*fimF* and *fimH*), StcE metalloprotease (*stcE*), and EhxA enterohemolysin (*EhxA*).

**Table 1:** Changes in gene expression for the main virulence factors in STEC.

Gene	Protein - Virulence factor	Function	Fold change STEC & EA SP	Fold change STEC & CW SP
				
***ler***	Ler	Master regulator LEE island	3.9	3.1
***eae***	Intimin	Adhesin	-1.2	-1.5
***tir***	Tir	Intimin receptor	1.1	-1.4
***lpfA***	LpfA – Lpf fimbriae	Fimbrial subunit	17.7	1.6
***lpfB***	LpfB – Lpf fimbriae	Chaperone	4.1	-1
***lpfD***	LpfD – Lpf fimbriae	Fimbrial subunit	2.7	1.6
***lpfE***	LpfE – Lpf fimbriae	Fimbrial subunit	2.9	1.4
***fliC***	FliC - flagellin	Fimbrial subunit	1.8	1.9
***toxB***	Toxin B	Expression and protein secretion	1.7	1.1
***stx2a***	Stx2 subunit A – Shiga toxin 2	Translation inhibition	-4.0	-1.4
***stx2b***	Stx2 subunit B – Shiga toxin 2	Binding to cellular receptor	-2.6	-1.1
***fimA***	FimA - Type I fimbriae	Fimbrial subunit	1.0	-1.6
***fimF***	FimF - Type I fimbriae	Fimbrial subunit	2.7	-1.3
***fimH***	FimH - Type I fimbriae	Fimbrial subunit - Adhesin	2.6	-1.3
***espA***	EspA – Type III secretion system	Fimbrial subunit	-2.1	-2.2
***espP***	EspP -Type III secretion system	Serine protease	1.3	-1.1
***hcp***	Hcp – Type III secretion system	Estructural subunit	1.3	1.2
***ehxA***	EhxA	Enterohemolysin	2.4	-1.0
***stcE***	StcE	Metalloprotease	3.4	1.5

STEC strain was incubated with the supernatant obtained from *E. albertii* (EA SP), *C. werkmanii* (CW SP), or M9 media (control) and gene expression level was measured by RNA-seq. Genes up-regulated (fold change ≥ 2) or down-regulated (fold change ≤ -2) are highlighted in blue and red, respectively.

For EAEC, an increase in 8.1% of genes coding for proteins associated with secretion systems and a 13.2% increase in genes coding for transcriptional regulators were found. Additionally, we observed a decrease in 8.5% of genes coding for proteins involved in adherence, 9.7% of genes coding for secretion systems protein and 52.2% of genes involved in EAEC motility (**Figure 1B**). Specifically, we found that incubation with EA supernatant produced a two-fold increase in expression of Pet toxin gene and a decrease in gene coding for flagellin (**Table 2**).

**Table 2:** Changes in gene expression for the main virulence factors in EAEC.

Gene	Protein - Virulence factor	Function	Fold change EAEC & EA SP	Fold change EAEC & CW SP
***aggR***	AggR	Master Regulator	1.4	1.5
***aafA***	AafA subunit (AAF fimbriae)	Adhesin protein	-1.5	1.1
***pic***	Pic: Protein Involved in Colonization	Mucinase	1.2	1.3
***aat***	ABC Transporter Protein	dispersin transporter	1.1	1.1
***pet***	Pet: Plasmid encoded toxin	serin-protease toxin	2.0	3.5
***lpfA***	LpfA subunit (LPF fimbriae)	Fimbrial subunit	1.6	1.8
***fliC***	FliC - flagellin	flagellar subunit	-8.7	-2.0
***fimC***	FimC – type I fimbriae	chaperone	1.3	2.2
***fimF***	FimF – type I fimbriae	Fimbrial subunit	-1.1	-1.8
***fimH***	FimH – type I fimbriae	Fimbrial subunit - Adhesine	1.3	1.5
***hlyE***	HlyE	Hemolysin	-1.3	1.7
***irp-1***	IRP-1: Iron regulatory protein	Translation regulator	-1.5	-1.7
***ag43***	Antigen 43	Adhesin	1.1	1.7
***hilA***	HilA	Transcriptional regulator	-1.1	1.1

EAEC strain was incubated with the supernatant obtained from *E. albertii* (EA SP), *C. werkmanii* (CW SP), or M9 media (control) and gene expression level was measured by RNA-seq. Genes up-regulated (fold change ≥ 2) or down-regulated (fold change ≤ -2) are highlighted in blue and red, respectively.

When STEC and EAEC prototype strains were incubated with CW SP, we observed that ~20% of genes changed their expression. In STEC, 570 genes were upregulated (10.7%) and 576 genes were downregulated (10.8%), and for EAEC, 527 genes were upregulated (10.2%) and 449 genes were downregulated (8.7%) (**Supplementary Table 2** and **Supplementary Figures 1C, D**). For genes involved in STEC pathogenicity, we observed an upregulation in more than 10% of genes coding for the transcriptional regulator (18.3%), toxins and toxin/antitoxin system (10%), and flagellar proteins (20%) (**Figure 1C**). Specifically, upregulation of gene coding for the master regulator Ler (*ler gene*) was observed in the presence of CW SP (**Table 1**). When EAEC was incubated with CW SP, there was an 11% increase in genes coding for flagellar proteins and transcriptional regulators, and a 7.1% increase in genes coding for toxins (**Figure 1D**). In the analysis of virulence factor expression, we observed an increase in expression of the genes coding Pet toxin and FimC fimbrial protein, along with a decrease in the gene coding for flagellin (**Table 2**).

To validate these results, RT-PCR analysis was performed on three genes involved in virulence for each DEC pathotype. For STEC, we evaluated the expression of genes coding for the master regulator of virulence Ler (*ler* gene), the LpfA fimbrial subunit responsible for bacterial adherence (*lpfA* gene) and Shiga toxin (*stx2* gene). Comparing with RNA-seq data, we obtained similar results, identifying an increase in Ler expression after incubation with EA or CW SP. We also confirmed an increase in *lpfA* gene expression and a decrease in *stx2* gene expression when STEC was incubated with EA SP. For EAEC, we evaluated the expression of genes coding for the master regulator AggR (*aggR* gene), AafA fimbrial subunit (*aafA* gene), and Pet toxin (*pet* gene). We confirmed an increase in Pet toxin expression after bacterial incubation with EA or CW SP, whereas the expression of genes coding for AggR and AafA did not change (**Table 3**).

**Table 3:** Confirmation RNA-seq results by RT-PCR.

	Gene	Virulence factor	Fold change EA SP(RT-PCR)	Fold change EA SP(RNAseq)	Fold change CW SP(RT-PCR)	Fold change CW SP(RNAseq)
**STEC**	*ler*	Ler, Master regulator LEE island	**6.3**	3.9	**3.4**	3.1
	*lpfA*	LpfA, fimbrial subunit Long Polar Fimbriae	**38.3**	17.7	**2.1**	1.6
	*stx2*	Shiga Toxin	**-1.7**	-2.6	**1.5**	-1.1
* *	* *					
**EAEC**	*aggR*	AggR, Master Regulator	**1.3**	1.4	**1.5**	1.5
	*aafA*	AafA, fimbrial subunit Aggregative Adherence Fimbriae	**-1.2**	-1.5	**-1.2**	1.1
	*pet*	Pet, serin-protease toxin	**13.0**	2.0	**23.0**	3.5

Fold change, compared to control condition, for the main virulence factors in STEC and EAEC after incubation with EA SP or CW SP. Genes up-regulated (fold change ≥ 2) are highlighted in blue.

### Higher IL-8 secretion by cells infected with STEC and EAEC in the presence of EA supernatant

**Figure 2:**
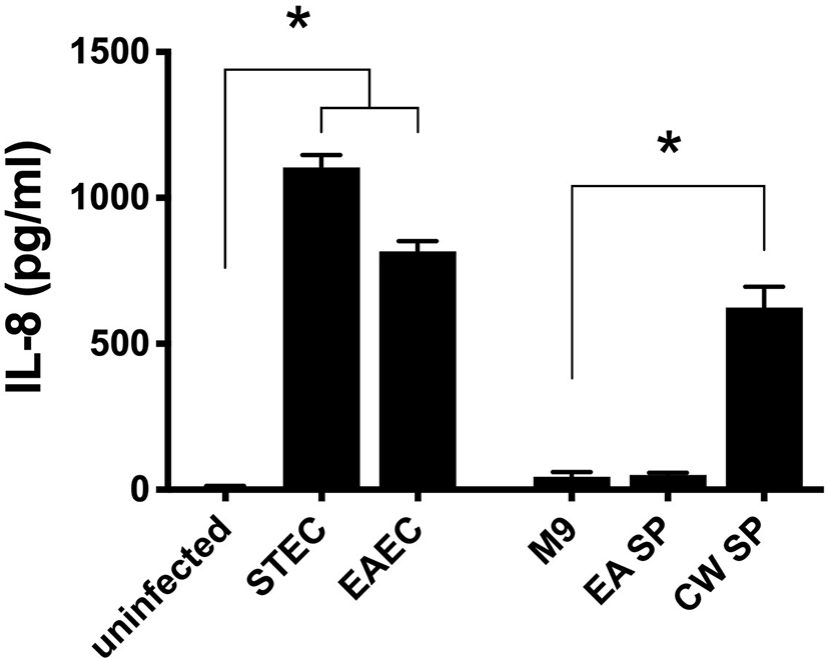
Induction of IL-8 secretion by bacteria used in this study. Intestinal cells in culture (T84 cells) were infected with STEC or EAEC or incubated with the supernatant obtained from the overnight growth of *E. albertii* (EA SP) or *C. werkmanii* (CW SP) in M9 medium. As a negative control, cells were incubated with DMEM medium (uninfected) or M9 medium (M9). Three hours post-infection/incubation, the level of IL-8 secretion was evaluated by ELISA. Graphed data are the mean of one representative experiment performed in triplicate, with the error bars indicating standard deviation. *p < 0.05 compared to control condition.

**Figure 3:**
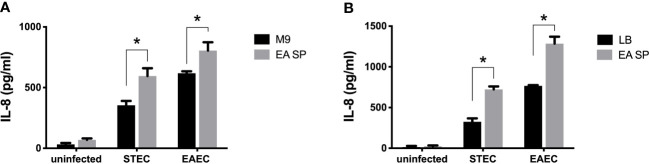
Induction of IL-8 secretion by DEC pathotypes in the presence of EA SP. T84 cells were infected with STEC or EAEC in the presence of the supernatant obtained from the overnight growth of *E*. *albertii* (EA SP) in M9 **(A)** or LB medium **(B)**. As a negative control, we used uninfected cells incubated with EA SP or with the medium used to prepare the supernatant. Three hours post-infection, the level of IL-8 secretion was evaluated by ELISA. Graphed data are the mean of one representative experiment performed in triplicate, with the error bars indicating standard deviation. *p < 0.05 by ANOVA and multiple comparison analysis.

To evaluate if the changes in virulence gene expression described above have an effect on DEC pathogenesis, we studied the ability of STEC and EAEC to induce IL-8 secretion by epithelial intestinal cells in the presence of EA or CW supernatant. As a preliminary experiment, we incubated intestinal cells with the supernatant obtained from EA or CW growth in M9 medium and measured the level of secreted IL-8 by ELISA. Interestingly, when cells were incubated with CW SP but not EA SP, we observed an increase in the IL-8 secretion by T84 cells compared with the IL-8 levels observed in the absence of these supernatants (**Figure 2**). For this reason, we decided to continue this study by evaluating only the effect of EA SP on DEC pathogenesis. We infected T84 cells with STEC or EAEC in the presence or absence of EA SP (prepared in M9 medium). Surprisingly, we found an increase in IL-8 secretion 3 h post-infection in cells infected with STEC or EAEC in the presence of EA SP compared to cells infected with DEC pathotypes that were not incubated with these supernatants (**Figure 3A**). Similar observations were found when the EA supernatant was prepared in LB medium (**Figure 3B**).

### Supernatants obtained from clinical *E. albertii* strains induce higher secretion of IL-8 by cells infected with STEC and EAEC

**Figure 4:**
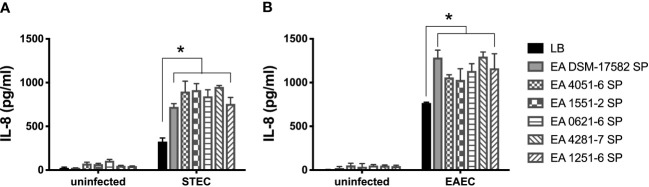
Induction of IL-8 secretion by DEC pathotypes in the presence of EA SP from clinical strains. T84 cells were infected with STEC **(A)** or EAEC **(B)** in the presence of the supernatant obtained from five clinical *E*. *albertii* strains (4051-6; 1551-2; 0621-6; 4281-7; 1251-6) and a reference strain (DSM-17582). We used uninfected cells incubated with the different EA SP as a negative control. Three hours post-infection, the level of IL-8 secretion was evaluated by ELISA. Graphed data are the mean of one representative experiment performed in triplicate, with the error bars indicating standard deviation. *p < 0.05 by ANOVA and multiple comparison analysis.

To determine if the increase in IL-8 secretion is associated with the incubation of STEC or EAEC with supernatant obtained from a commercial *E. albertii* strain, we quantified the level of IL-8 secreted by cells infected with STEC or EAEC in the presence of supernatants obtained from five clinical strains. In STEC and EAEC, we found a significantly higher secretion of IL-8 when DEC pathotypes were incubated with different EA supernatants compared to the IL-8 levels induced by these DEC pathotypes alone. None of the EA supernatants obtained from clinical strains was able to induce IL-8 secretion (**Figure 4**).

### *E. albertii* have no effect on growth and adherence to cells of STEC or EAEC

**Figure 5:**
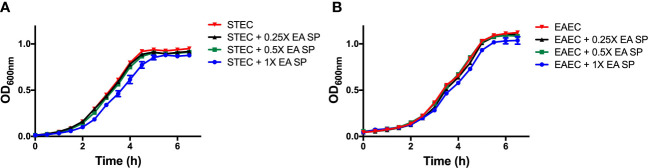
STEC and EAEC growth curve in the presence of different concentrations of EA SP. Overnight cultures from STEC **(A)** and EAEC **(B)** were diluted in DMEM HG medium with different concentrations of EA SP. These bacteria were grown for 6 h at 37°C and optical density at 600 nm (OD_600nm_) was measured every 30 min. Results are expressed as the means ± the standard errors for one of three experiments done in triplicate. Difference between groups were tested by ANOVA and multiple comparison test.

**Figure 6:**
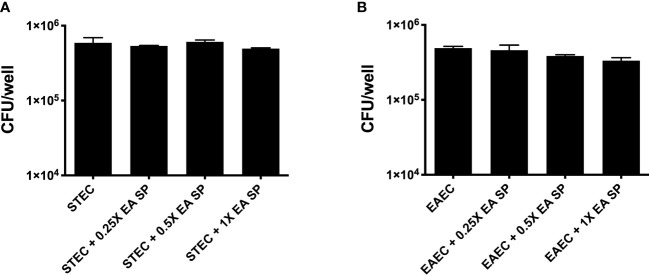
Adherence of STEC and EAEC to intestinal cells in the presence of EA SP.T84 cells were infected with STEC **(A)** or EAEC **(B)** in the presence of different concentrations of the supernatant obtained from the overnight growth of *E. albertii* (EA SP). After 3 h of infection, the number of adherent bacteria was determined by counting colony-forming units (CFU) in LB agar plates. Results are expressed as the means ± the standard errors for one of three experiments done in triplicate. Difference between groups were tested by ANOVA and multiple comparison test.

Next, we evaluated if the IL-8 secretion increase could be explained by a higher growth rate or a higher bacterial adhesion of DEC pathotypes during infection. First, the effect of EA SP, at different concentrations, on the growth of STEC 86-24 and EAEC 042 was studied. Compared to STEC or EAEC growth in LB alone, we found no difference in DEC growth at different concentrations of EA SP used (**Figure 5**). Also, the effect of EA supernatant on the ability of STEC and EAEC to adhere to epithelial intestinal cells was evaluated. We observed no difference in adherence of STEC or EAEC to T84 cells (**Figure 6**).

### Lpf fimbriae in STEC participate in the induction of IL-8 by cells infected with ECD in the presence of the *E. albertii* supernatant

**Figure 7:**
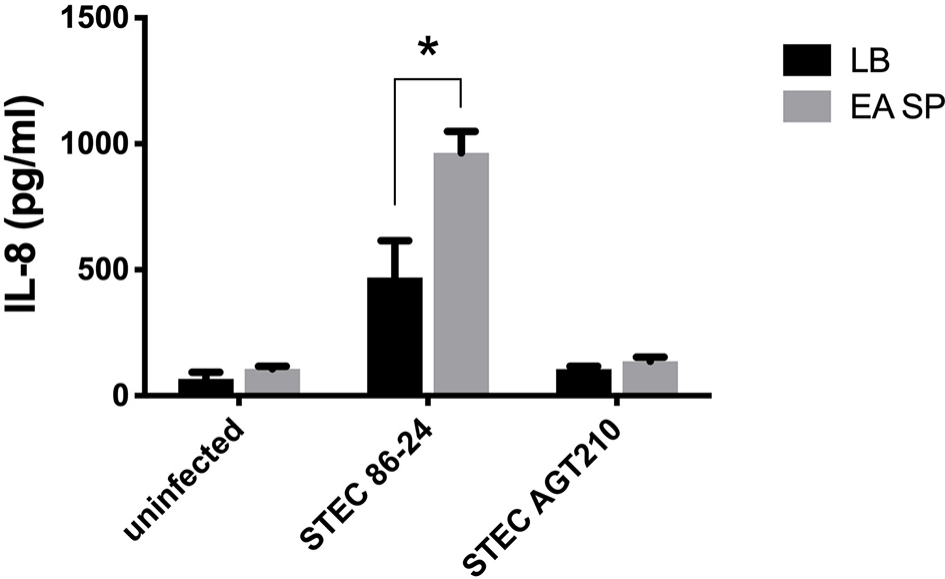
Induction of IL-8 secretion by STEC 86-24 and STEC AGT210 in the presence of EA SP. T84 cells were infected with STEC wild-type strain or STEC double mutant in Lpf fimbriae (AGT210) in the presence of the supernatant obtained from the overnight growth of *E. albertii* (EA SP). As a negative control, we used uninfected cells incubated with EA SP or with LB, the medium used to prepare the supernatant. Three hours post-infection, the level of IL-8 secretion was evaluated by ELISA. Graphed data are the mean of one representative experiment performed in triplicate, with the error bars indicating standard deviation. *p < 0.05 by ANOVA and multiple comparison analysis.

We previously described long polar fimbriae in STEC as being associated with the induction of an inflammatory response in T84 cells (Vergara et al., 2014). As we observed an increase in the expression of the *lpf* operon in the RNA-seq experiment, we evaluated the role of these fimbriae in the increase of IL-8 secretion in the presence of EA supernatant. For this purpose, T84 cells were infected with STEC or with a STEC mutant strain in Lpf fimbriae (STEC AGT210) in the presence or absence of EA SP, and the induction of IL-8 secretion was evaluated. In the Lpf mutant incubated with EA SP, we found no increase in IL-8 secretion as we observed for the 86-24 wild-type strain (**Figure 7**).

### Secretome of *E. albertii* induces a higher secretion of Pet toxin in EAEC

**Figure 8:**
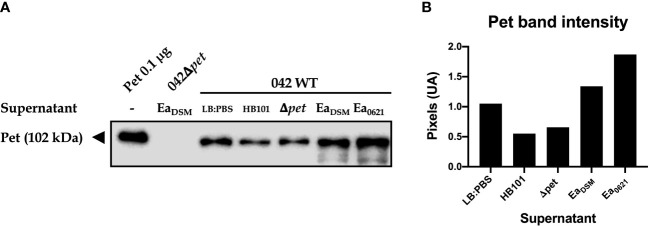
Pet secretion by EAEC 042 in the presence of EA SP. EAEC 042 wild-type strain was incubated with the supernatant obtained from the *E*. *coli* HB101 strain (HB101), EAEC strain mutant in Pet toxin (Δ*pet*), *E*. *albertii* reference strain DSM-17582 (Ea_DSM_), and *E*. *albertii* clinical strain 0621-6 (Ea_0621_). As a control, EAEC 042 was incubated with the same medium used to prepare the supernatants (LB : PBS). Also, the EAEC mutant strain in Pet toxin was incubated with the supernatant obtained from Ea_DSM_. The Pet toxin was precipitated with TCA and detected by Western blot, with a rabbit antibody against Pet **(A)**, and the protein band intensity was determined **(B)**.

As we observed that Pet toxin gene expression was increased in the presence of EA SP, we evaluated the effect of this supernatant on Pet toxin secretion by EAEC 042. Additionally, the effects of the supernatant from *E. coli* HB101 and EAEC 042*Δpet* on Pet toxin secretion were evaluated. Pet was detected in TCA-precipitated supernatant by Western blotting (pixel density) and purified Pet toxin was used as positive control. In TCA-precipitated supernatant from EAEC incubated with LB : PBS (control), a 104-kDa protein band was detected corresponding to Pet toxin (Betancourt-Sanchez and Navarro-Garcia, 2009). We noted that EA SP is able to induce a higher Pet toxin secretion in the EAEC 042 culture compared to the control condition. Unlike EA SP, supernatants obtained from *E. coli* HB101 and 042*Δpet* did not induce a higher Pet toxin secretion compared to the control (**Figure 8**). Moreover, the effect of the supernatant obtained from a clinical EA strain (strain 0621) on Pet toxin secretion was evaluated. Surprisingly, this supernatant was able to induce a higher secretion of Pet toxin than that observed with EA SP (**Figure 8**).

## Discussion

DEC pathotypes have evolved from commensal *E. coli*, acquiring virulence traits and sensing mechanisms to express virulence factors at the right time and place for the successful colonization of intestinal cells (Kitamoto et al., 2016). Among the environmental conditions, gut microbiota composition might play a role in the regulation of pathogenic mechanisms (Tsolis and Baumler, 2020). In this study, we provided experimental evidence that indicative species of gut microbiota associated with DEC infection might regulate the expression of virulence genes in STEC and EAEC.

It is well known that changes in microbiota composition occur in diarrhea (Pop et al., 2014; Samb-Ba et al., 2014; Schubert et al., 2014; Pop et al., 2016), and this could have an impact on the environmental conditions present at the infection sites (Vogt et al., 2015; Gallardo et al., 2020). In a previous study, we identified indicative bacterial species of gut microbiota associated with DEC-positive diarrhea stool samples from children under 5 years of age (Gallardo et al., 2017). In this work, we evaluated the role of two of these indicative species, *Citrobacter werkmanii* (CW) and *Escherichia albertii* (EA), in the infection process of the DEC pathotypes STEC and EAEC.

Considering that pathogenic bacteria can use microbiota-derived sources of carbon and nitrogen as nutrient and regulatory signals to promote their own growth and virulence (Baumler and Sperandio, 2016), our first approach was to evaluate the effect of the supernatant of *C. werkmanii* and *E. albertii* on the gene expression of virulence factor of STEC and EAEC by RNA-seq experiments. In STEC, an increase was observed in the expression of the gene coding for the master regulator of LEE pathogenicity island (*ler* gene) and virulence genes regulated by Ler (**Table 1**), a situation that could be indicative of a positive regulation of STEC virulence *in vitro* (Honda et al., 2009; Garcia et al., 2012). A similar situation was previously reported, where, after incubating STEC with *B. thetaiotaomicron* or *E. faecalis*, both bacteria of gut microbiota present in healthy people, there was an increase in *ler* gene expression and several virulence genes in STEC (Curtis et al., 2014; Iversen et al., 2015). Along with this information, we observed an increase in the genes coding for different subunits of Lpf fimbriae (genes *lpfA*, *lpfB*, *lpfD*, and *lpfE*), a relevant virulence factor for STEC adherence to intestinal cells (Torres et al., 2002). For EAEC, we found a downregulation of the gene coding for the protein FliC, belonging to flagellin, which can induce IL-8 secretion in intestinal cells by interacting with the cellular receptor TLR5 (Khan et al., 2004). The regulation of the *fliC* gene expression by metabolites has been previously reported. *E. coli* incubation with short-chain fatty acids (SCFAs) at 20 mM produced an increase in FliC protein levels along with a higher bacterial motility (Tobe et al., 2011); however, colonic levels of SCFA (65 mM) downregulated genes associated with motility (*fliC*) in 11 out of 15 *E. coli* strains tested (Zhang et al., 2020). Furthermore, we found an increase in the expression of the gene coding for Pet toxin, an autotransporter-secreted protein with serine protease activity that has been described as having enterotoxic and cytotoxic effects (Navarro-Garcia et al., 2010). It has also been reported that Pet can induce proinflammatory cytokine secretion as IL-8 and TNF-α in macrophages (Rocha-Ramirez et al., 2016). Together, these results suggest that secreted components by indicative bacteria from intestinal microbiota of DEC-positive diarrhea can regulate the expression of virulence factors in STEC and EAEC and suggest a higher virulence of these DEC pathotypes in the presence of EA or CW SP.

Previous studies have shown that the microbiota shapes the chemical environment of the gut, producing metabolites that have an effect on the host and other microbes (reviewed in Vogt et al., 2015). It has been reported that spent culture medium from human fecal microbiota inhibits virulence gene expression in enteropathogens; for example, in STEC, the spent culture medium repressed the expression of the *stx2* gene (de Sablet et al., 2009). Also, isolated *Peptostreptococcus* sp. and *Lactobacillus* sp. from human fecal samples secreted unidentified metabolites that inhibited *Vibrio cholerae* growth (Silva et al., 2001). Microbiota could also have effects on the host, such as modulating immune functions or altering the expression of host receptors for bacterial toxins (Vogt et al., 2015). For example, SCFAs (generated by fermentation of dietary fibers by intestinal commensal microbiota) can elicit the production of cytokines, including TNF-a and IL-6, and chemokines, such as CXCL1 and CXCL2, by colonic epithelial cells, which induce a protective immune response against pathogens by promoting the recruitment of neutrophils and the development of Th17 cells (Kayama and Takeda, 2020). When we studied the effect of EA and CW SP on the ability to induce IL-8 secretion by epithelial intestinal cells, we found that CW SP can induce IL-8 secretion without the presence of DEC pathotypes; no IL-8 secretion was observed when EA SP was added to T84 cells (**Figure 2**). A previous study using supernatants from four potentially probiotic *E. coli* strains showed that one of them can induce IL-8 secretion on intestinal cells in culture, and this could be attributable to differences in bacterial surface molecules (Zimmermann et al., 2018). Cell extract from *E. coli* Nissle can also induce IL-8 production in a dose-dependent way, suggesting that secretory proteins, virulent or not, may play a role in IL-8 production (Lammers et al., 2002). This evidence suggests that some bacterial metabolites present in CW SP can activate this signaling pathway; however, the mechanism involved in this process has not been discovered yet. For this reason, we decided to evaluate the effect of EA SP and not CW SP on the ability of STEC and EAEC to induce IL-8 secretion on intestinal epithelial cells.

We found that incubation of EA SP with EAEC or STEC induced a higher secretion of IL-8 compared to the control condition (**Figure 3**). Interestingly, this increase in IL-8 secretion can also be observed with the supernatant obtained from five clinical strains of *E. albertii* (**Figure 4**), which suggests that the effect is associated with *E. albertii* species and not with a particular strain. The secretion of IL-8 by intestinal cells creates a chemotactic gradient that promotes the migration of neutrophils to the infection site (Godaly et al., 1997). It has been reported that the presence of neutrophils at infection sites can favor EAEC colonization, producing an increase in adherent bacteria to cells in culture and promoting pathogenesis (Boll et al., 2012). It has also been reported that pathogenic bacteria, by eliciting inflammation, can alter the intestinal environment and create a fresh niche of respiratory nutrient suitable for their expansion in the gut (Baumler and Sperandio, 2016). Considering that different supernatants from *E. albertii* cannot by themselves induce an inflammatory response, the effect observed could be associated with a higher expression of virulence factors in DEC pathotypes that are capable of causing an inflammatory response in intestinal cells.

Adherence to intestinal cells is a key step in DEC pathogenesis, a process mediated by bacterial adhesins. For STEC and EAEC, several adhesins have been described; some with the ability to participate in other steps of pathogenesis, such as the induction of an inflammatory response in infected cells or tissues (Harrington et al., 2005; Boll et al., 2012; Farfan et al., 2013). Of the genes coding for proteins involved in adherence, RNA-seq data revealed that 35% and 8.5% of these genes were upregulated for STEC and EAEC, respectively (**Figures 1A, B**). Surprisingly, we did not observe any differences in the adherence of STEC and EAEC to T84 cells in the presence of EA SP (**Figure 6**). These observations can be explained by the ability of STEC and EAEC to adhere to intestinal cells in large numbers (approximately 5 × 10^5^ CFU/well). The upregulation in the expression of genes found in RNA-seq experiments was likely insufficient to significantly increase the adherence to cells. Further experiments to correlate the RNA-seq data and the adherence of STEC and EAEC observed, such as protein levels of adhesins or their expression on the bacterial surface, are needed.

Previous experiments from our group have reported that Lpf is also one of the factors that mediate adherence of STEC to intestinal cells and the inflammatory response through NF-kB pathway activation, caused by this DEC pathotype (Torres et al., 2002; Farfan et al., 2013; Vergara et al., 2014). To evaluate the role of Lpf in the induction of IL-8 secretion by STEC incubated with EA SP, we used a double mutant in the *lpfA1* and *lpfA2* genes (strain AGT210), which adhered similarly to the wild-type strain STEC 86-24, but induced a reduced amount of IL-8 secretion by infected T84 cells (Farfan et al., 2013). We found that strain AGT210 did not present an increase in IL-8 secretion in the presence of EA SP (**Figure 7**), suggesting that Lpf fimbriae might be participating in the increase of IL-8 observed in the presence of EA SP. As we discussed above, we did not note a higher STEC adhesion to intestinal cells (**Figure 6**). Receptors involved in the recognition of Lpf fimbriae and responsible for the activation of an inflammatory response have not been discovered yet. Previous studies have explored possible receptors for these fimbriae, and found that Lpf is capable of binding to extracellular matrix proteins such as fibronectin, laminin, and collagen IV (Farfan et al., 2011), but its participation in the activation of an inflammatory response by these bacteria has not been explored. More studies are needed to correlate the induction gene expression of Lpf fimbriae coding genes, the expression of these fimbriae at the surface of bacteria, and the effect on adherence to cells as well as the inflammatory response induced by this adhesin. Pet toxin is a key virulence determinant in EAEC pathogenesis (Villaseca et al., 2000). In this study, we found that EA SP induced a higher secretion of Pet toxin by EAEC than the control conditions (**Figure 8**). Considering this toxin has been associated with inducing the secretion of inflammatory cytokines (Rocha-Ramirez et al., 2016), we suggest that Pet could be participating in the increase in IL-8 observed. This increase in Pet toxin secretion was even higher in the presence of the supernatant obtained from clinical EA strain (*E. albertii* 0621). A similar situation was noted in a previous study with the metabolite succinate, originally detected in the supernatant from *B. tethaiotaomicron*, which is responsible for the increase in virulence factor EspA in STEC (Curtis et al., 2014). Further studies evaluating the role of Pet toxin in EAEC infection to cells in the presence of EA SP are needed.

In this work, our results have shown that the supernatant from *E. albertii*, a bacterium from intestinal microbiota associated with DEC infections, can induce changes in gene expression for two DEC pathotypes, including regulators of virulence, toxins, and fimbriae. These changes had an effect on the inflammatory response caused by these bacteria, causing an increase in IL-8 levels secreted by intestinal cells after the infection with STEC or EAEC. Together, our data suggest that metabolites secreted by *E. albertii* modulate the virulence of STEC and EAEC, producing changes in gene expression and affecting the inflammatory process caused by DEC pathotypes. Further experiments to identify the metabolites in the EA SP might be useful to enhance our knowledge of STEC and EAEC pathogenicity, and of the mechanism and signaling molecules involved in the crosstalk between the microbiota and enteropathogens.

## Data availability statement

The RNA-seq data discussed in this publication have been deposited in NCBI's Gene Expression Omnibus (Edgar et al., 2002) and are accessible through GEO Series accession number GSE197797, https://www.ncbi.nlm.nih.gov/geo/query/acc.cgi?acc=GSE197797).

## Author contributions

MI performed the experiments, data analysis, interpreted the data and participated in manuscript writing. JL performed the experiments, data analysis. RV participated in study design and data analysis. PG participated in data analysis. JO participated in data analysis. MF participated in study design, interpretation of data, manuscript writing and final approval of the manuscript. All authors contributed to the article and approved the submitted version.

## Acknowledgments

This work was supported by an ANID grant 1200994 to MF. MI and PG were supported by a doctoral fellowship from ANID. We thank Dr. Tânia A. T. Gomes (Federal University of Sao Paulo, Brazil) for the *E. albertii* clinical strains and Dr. Fernando Navarro-Garcia (CINVESTAV, Mexico) for help and guidance with the Pet secretion experiments.

## Conflict of interest

The authors declare that the research was conducted in the absence of any commercial or financial relationships that could be construed as a potential conflict of interest.

## Publisher’s note

All claims expressed in this article are solely those of the authors and do not necessarily represent those of their affiliated organizations, or those of the publisher, the editors and the reviewers. Any product that may be evaluated in this article, or claim that may be made by its manufacturer, is not guaranteed or endorsed by the publisher.

## Supplementary material

Supplementary Figure 1 Changes in gene expression for DEC pathotypes incubated with EA or CW SP. Reference strains of STEC **(A, C)** and EAEC **(B, D)** were incubated with M9 medium (control) or supernatant from EA **(A, B)** or CW **(C, D)**. RNA extraction and analysis by RNA-seq was performed. Changes in gene expression, compared to the control condition, were colored according to the change observed; genes upregulated are in blue, genes downregulated are in red, and genes with no change compared to the control are in gray.

Supplementary Table 1 Primers used in this study.

Supplementary Table 2 Changes in gene expression determined by RNA-seq, for STEC and EAEC after incubation with EA or CW SP.
