# Supplementary figures and images for: Bacteria from gut microbiota associated with diarrheal infections in children promote virulence of Shiga toxin-producing and enteroaggregative Escherichia coli pathotypes
Source: Front Cell Infect Microbiol. 2022 Aug 9;12:867205. doi: 10.3389/fcimb.2022.867205 (PMC9396624; doi:10.3389/fcimb.2022.867205)

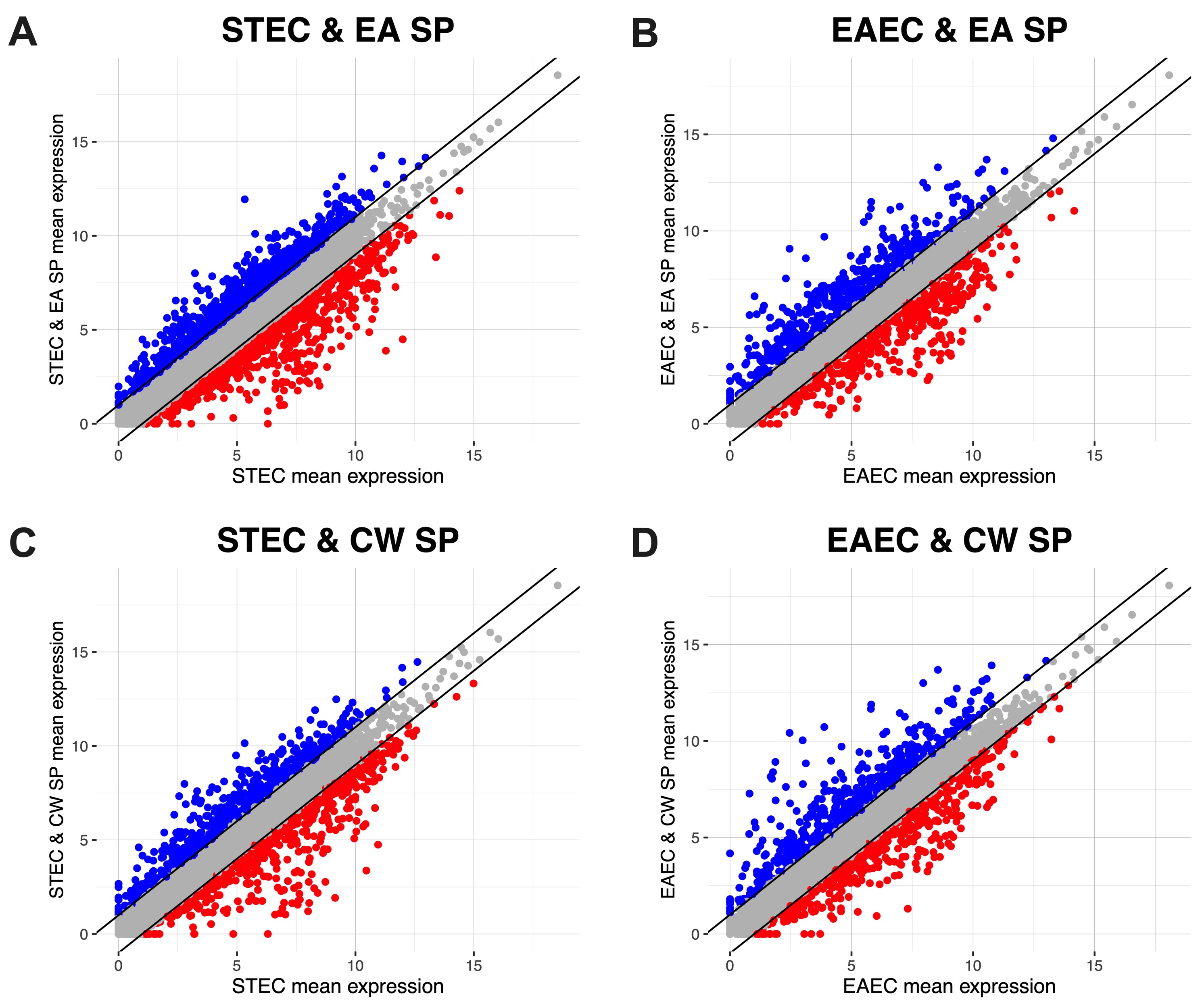

Supplement: Supplementary Figure 1 — Changes in gene expression for DEC pathotypes incubated with EA or CW SP. Reference strains of STEC (A, C) and EAEC (B, D) were incubated with M9 medium (control) or supernatant from EA (A, B) or CW (C, D). RNA extraction and analysis by RNA-seq was performed. Changes in gene expression, compared to the control condition, were colored according to the change observed; genes upregulated are in blue, genes downregulated are in red, and genes with no change compared to the control are in gray. [file Image_1.jpg]
