# Supplementary material for: Bacteria from gut microbiota associated with diarrheal infections in children promote virulence of Shiga toxin-producing and enteroaggregative Escherichia coli pathotypes
Source: Front Cell Infect Microbiol. 2022 Aug 9;12:867205. doi: 10.3389/fcimb.2022.867205 (PMC9396624; doi:10.3389/fcimb.2022.867205)
Supplement: Supplementary Table 1 — Primers used in this study. [file Table_1.docx]

Supplementary Table 1.

|  | **Gene** | **Sequence** | **Reference** |
| --- | --- | --- | --- |
| **STEC** | *ler* | F: 5`- CGA CCA GGT CTG CCC TTC T -3`  R: 5`- GCG CGG AAC TCA TCG AAA -3` | Walters and Sperandio, 2006 |
|  | *lpfA* | F: 5`- GGC ACG GTT AAA TTT ACA GGC -3`  R: 5`- TGA CCC AAC ACA ACT TCC TG -3` | This study |
|  | *stx2* | F: 5`-GCT GGA ATC TGC AAC CGT TAC T -3`  R: 5`-CAC GAA TCA GGT TAT GCC TCA GT -3` | Qu et al., 2014 |
| **EAEC** | *aggR* | F: 5`- CAG AAT CGT CAG CAT CAG CTA CA -3`  R: 5`- AAG GAT GCC CTG ATG ATA ATA TAC G -3’ | Qu et al., 2014 |
|  | *aafA* | F: 5`-ATT CAC TCT GGC CTC TCC TAG GT -3`  R: 5`-ACT TCA TAT AGG CCT GGT CGT A -3` | Hinthong et al., 2015 |
|  | *pet* | F: 5`-GTG GTG CCT ATG CCG TAA CC-3`  R: 5`-CAG CCC CTC TTG TTT CCA CG-3` | Betancourt-Sanchez and Navarro-Garcia, 2009 |
| **Reference genes** | *rpoA* | F: 5`- GCG CTC ATC TTC TTC CGA AT -3`  R: 5`- CGC GGT CGT GGT TAT GTG -3` | Walters and Sperandio, 2006 |
|  | *16S* | F: 5`- AAT AAA TCA TAA ACT CCT ACG GGA GGC AGC AGT -3`  R: 5`- AAT AAA TCA TAA CCT AGC TAT TAC CGC GGC TGC T– 3` | Brukner et al., 2015 |
