# Supplementary material for: Bacteria from gut microbiota associated with diarrheal infections in children promote virulence of Shiga toxin-producing and enteroaggregative Escherichia coli pathotypes
Source: Front Cell Infect Microbiol. 2022 Aug 9;12:867205. doi: 10.3389/fcimb.2022.867205 (PMC9396624; doi:10.3389/fcimb.2022.867205)
Supplement: Supplementary Table 2 — Changes in gene expression determined by RNA-seq, for STEC and EAEC after incubation with EA or CW SP. [file Table_2.docx]

Supplementary Table 2

|  | **STEC & EA SP** | **EAEC & EA SP** | **STEC & CW SP** | **EAEC & CW SP** |
| --- | --- | --- | --- | --- |
| **Increase** | 889 (16.7%) | 525 (10.1%) | 570 (10.7%) | 527 (10.2%) |
| **No change** | 3661 (68.7%) | 4225 (81.6%) | 4184 (78.5%) | 4201 (81.1) |
| **Decrease** | 780 (14.6%) | 427 (8.3%) | 576 (10.8%) | 449 (8.7%) |
| **Total** | 5330 | 5177 | 5330 | 5177 |
